# Design, Synthesis and Structure-Activity Relationships of Novel Diaryl Urea Derivatives as Potential EGFR Inhibitors

**DOI:** 10.3390/molecules21111572

**Published:** 2016-11-18

**Authors:** Nan Jiang, Yanxin Bu, Yu Wang, Minhua Nie, Dajun Zhang, Xin Zhai

**Affiliations:** 1 Key Lab. of New Drugs Design and Discovery of Liaoning Province, School of Pharmaceutical Engineering, Shenyang Pharmaceutical University, 103 Wenhua Road, Shenhe District, Shenyang 110016, China; jiangnan@syphu.edu.cn (N.J.); yanxinbu@163.com (Y.B.); wangyubobby@163.com (Y.W.); 18604019493@163.com (M.N.); 2 Department of Chemsitry, Shenyang Medical College, 146 Huanghe North Street, Huanggu District, Shenyang 110034, China

**Keywords:** diaryl urea, 4-aminoquinazolinyl, synthesis, cytotoxicity, EGFR inhibitors

## Abstract

Two novel series of diaryl urea derivatives **5a**–**i** and **13a**–**l** were synthesized and evaluated for their cytotoxicity against H-460, HT-29, A549, and MDA-MB-231 cancer cell lines in vitro. Therein, 4-aminoquinazolinyl-diaryl urea derivatives **5a**–**i** demonstrated significant activity, and seven of them are more active than sorafenib, with IC_50_ values ranging from 0.089 to 5.46 μM. Especially, compound **5a** exhibited the most active potency both in cellular (IC_50_ = 0.15, 0.089, 0.36, and 0.75 μM, respectively) and enzymatic assay (IC_50_ = 56 nM against EGFR), representing a promising lead for further optimization.

## 1. Introduction

**Figure 1:**
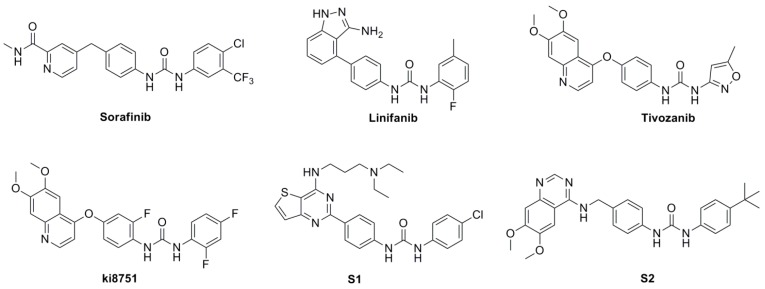
Structures of diaryl urea derivatives.

Recently, small molecular multiple targeted drugs have played a crucial role in cancer therapy due to their high efficiency and low toxicity. Diaryl urea derivative sorafenib [1], the first oral multikinase inhibitor targeted Raf and receptor tyrosine kinases (RTKs), has been applied for the treatment of advanced renal cell carcinoma (RCC) [2], unresectable hepatocellular carcinoma (HCC) [3], and differentiated thyroid carcinoma (DTC) [4]. It is reported that the lipophilic diaryl urea moiety served as a key structural fragment for binding with the hydrophobic pocket of the kinase domain through hydrogen bonds and hydrophobic interactions [5]. Subsequently, diverse diaryl urea derivatives were developed successively in the past decades, such as linifanib [6], tivozanib [7], and ki8751 [8] (Figure 1). Therein, thieno[3,2-*d*]pyrimidine derivative **S1** bearing diaryl urea moieties at the C-2 positon exhibited significant inhibition of tyrosine kinases, including c-Kit, orphan receptor tyrosine kinase (RET), and FLT3 for its prominent framework [9]. Similarly, the 4-aminoquinazolinyl skeleton, which competitively binds to the ATP binding pocket of intracellular kinase domains and blocks the conduction of downstream signaling networks mediated by tyrosine kinase, is also extensively used in the design of RTKs inhibitors (e.g., gefitinib, erlotinib and **S2**) [10,11].

**Figure 2:**
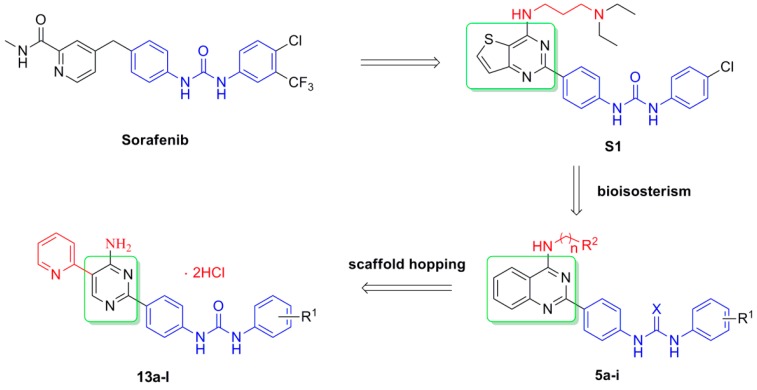
Design of target compounds.

In light of the abovementioned considerations, and as ongoing efforts to identify new potent antitumor agents, a novel series of 4-aminoquinazolinyl-diaryl urea derivatives **5a**–**i** were designed according to bioisosterism theory to achieve synergistic antitumor effects (Figure 2). In addition, a variety of substituents and aliphatic amino were introduced into the terminal phenyl group and C-4 position on the quinazoline ring to explore the influence of electronic and steric hindrance effect. Furthermore, another novel series of diaryl urea derivatives **13a**–**l** bearing a 5-pyridyl-4-aminopyrimidinyl motif were designed based on scaffold hopping principle, in the hope of attaining structural diversity and optimal cellular potency via improving water solubility. All compounds were synthesized and evaluated for their cytotoxicity against HT-29, H-460, A549, and MDA-MB-231 cancer cell lines. Additionally, enzymatic assay of the most active compound **5a** was also presented in this study.

## 2. Results and Discussion

### 2.1. Chemistry

**Scheme 1:**
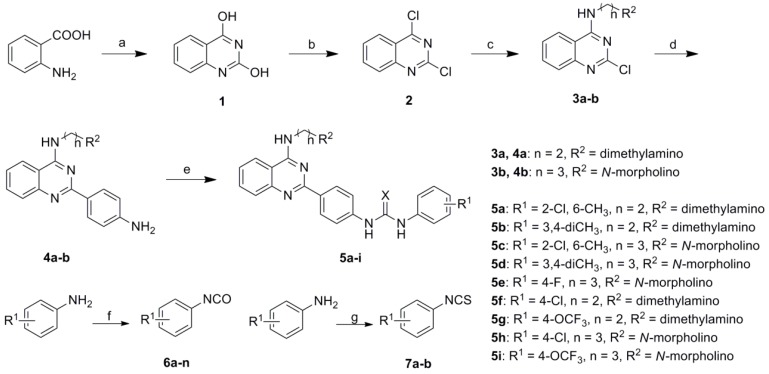
Synthesis of target compounds **5a**–**i**. *Reagents and conditions*: (**a**) urea, 160 °C, 12 h; (**b**) POCl_3_, DIPEA, 90 °C, 6 h; (**c**) NH_2_(CH_2_)_n_R^1^, THF, TEA, 30 °C, 15 min; (**d**) 4-aminophenylboronic acid pinacol ester, Na_2_CO_3_, Pd(PPh_3_)_2_Cl_2_, THF, N_2_, r.f., 5 h; (**e**) **6a**–**d** or **7a**–**b**, THF, 30 °C, 6 h; (**f**) BTC, 1,4-dioxane, r.f., 8–12 h; (**g**) (**i**) DABCO, CS_2_, toluene, r.t., 12 h; (**ii**) BTC, CHCl_3_, r.f., 1 h.

The synthesis of target compounds **5a**–**i** is depicted in Scheme 1. The synthesis of intermediates **3a**–**b** have been described in detail in our previous work [12], so the synthetic method is not listed here. [12]. A Suzuki reaction of **3a**,**b** with commercially available 4-aminophenylboronic acid pinacol ester under nitrogen protection provided the key intermediates **4a**,**b** [13]. A series of substituted aromatic isocyanates **6** and isothiocynates **7** were subsequently prepared by treating the corresponding arylamine with triphosgene (BTC) or 1,4-diazabicyclo[2.2.2]octane (DABCO) without further purification [14,15]. Eventually, target compounds **5a**–**i** were successfully obtained via the reaction of **4a**,**b** with corresponding **6** or **7** in THF at 30 °C, respectively.

**Scheme 2:**
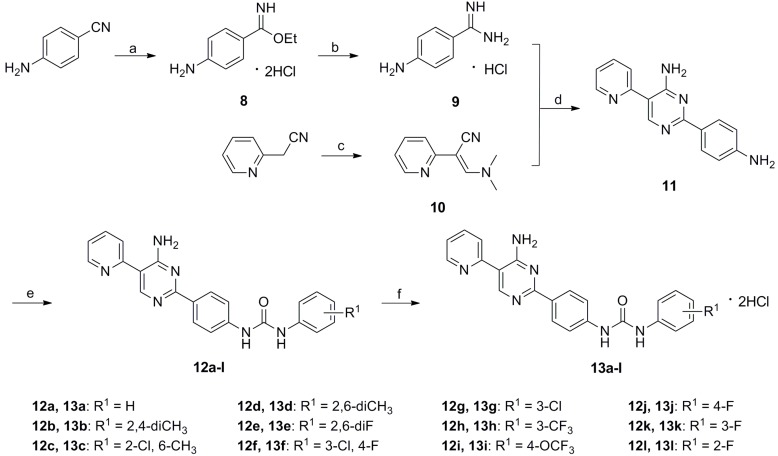
Synthesis of target compounds **5a**–**i**. *Reagents and conditions*: (**a**) HCl, EtOH-1,4-dioxane, 0 °C, 6 h, r.t., 48 h; (**b**) NH_3_, EtOH, 0 °C, 6 h, r.t., 24 h; (**c**) DMF-DMA, MeOH, 30 °C, 24 h; (**d**) Na_2_CO_3_, MeOH-H_2_O, 70 °C, 24 h; (**e**) **6c**–**n**, THF, 30 °C, 6 h; and (**f**) HCl-EtOH, CHCl_3_, 1 h.

The synthetic route of target compounds **13a**–**l** is outlined in Scheme 2. Dry HCl was bubbled into the mixture of 4-aminobenzonitrile in EtOH and 1,4-dioxane in an ice bath to generate compound **8**, which was further replaced with dry NH_3_ to afford intermediate **9** [16]. **10** was prepared via reaction of 2-(pyridin-2-yl)acetonitrile and excess *N*,*N*-dimethylformamide dimethyl acetal (DMF-DMA) at 30 °C for 24 h [17]. Condensation reaction of **9** and **10** in MeOH-H_2_O solution in the presence of Na_2_CO_3_ gave rise to intermediate **11** [18]. Subsequently, **11** condensed with corresponding aromatic isocyanate **6c**–**n** in a similar manner as described for compounds **5a**–**i** to furnish **12a**–**l**. Finally, **12a**–**l** were treated with 20%–30% hydrochloride ethanol solution to provide target compounds **13a**–**l**.

### 2.2. Biological Results and Discussion

For summarizing structure-activity relationships (SARs), all target compounds (**5a**–**i** and **13a**–**l**) were evaluated for their cytotoxicity against HT-29 (human colon cancer), H-460 (human lung cancer), A549 (human lung cancer), and MDA-MB-231 (human breast cancer) cell lines by 3-(4,5-dimethylthiazol-2-yl)-2,5-diphenyltetrazolium bromide (MTT) assay, taking sorafenib as the positive control. The results expressed as IC_50_ values (μM) are presented in Table 1 and Table 2.

As shown in Table 1, compounds **5a**–**i** demonstrated excellent activity towards four tested cell lines with IC_50_ values ranging from 0.089 to 5.46 μM, and seven of them were more active against tested cell lines than the positive control sorafenib. It was noticeable that all compounds exhibited prominent cytotoxicity against HT-29 superior to sorafenib, reflecting good selectivity for colon cancer. In general, antitumor potency was related to the amino group R^1^ on side chain, and dimethylamino was more effective than morpholinyl (**5a** vs. **5c**, **5b** vs. **5d**, **5g** vs. **5i**), which might be due to its smaller bulk and stronger hydrophilia. Additionally, the substituent R^2^ on the terminal phenyl ring exerted a major influence on pharmacological activity. Electrophilic groups are beneficial to the improvement of antitumor potency, as might be the reason of compounds **5a**, **5c**, **5f**, and **5h** with the Cl group showing eminent cytotoxicity. Conversely, a certain decrease in activity was observed once 3,4-(CH_3_)_2_ were introduced, such as compounds **5b** and **5d**. Interestingly, 2-CH_3_ and 6-Cl analogue **5a** showed best activity, with IC_50_ values of 0.15, 0.089, 0.36, and 0.75 μM, which were five- to 24-fold more potent than sorafenib respectively. The results indicated that steric hindrance in ortho-position was preferred.

To further verify the optimized structural skeleton and explore the SARs, pharmacological data of compounds **13a**–**l** bearing 4-aminopyrimidinyl moiety are illustrated in Table 2. Unexpectedly, the results were unsatisfied although multiple hydrophilic modifications were performed by introducing pyridine group and further salifying with hydrochloride. The contrast of antitumor potency between 4-aminoquinazoline and 4-aminopyrimidine derivatives suggested that further structural modification might have broken the binding affinity to intracellular kinase domains or switched the binding affinity to other targets, while the 4-aminoquinazolinyl moiety was an excellent skeleton for EGFR inhibitors. The activity toward EGFR kinase of compound **5a** was surprised with the IC_50_ value of 56 nM, indicating that 4-aminoquinazolinyl-diaryl urea derivatives **5a**–**i** were potent EGFR inhibitors (Table 3). Efforts to identify its mechanisms of action and further optimization are ongoing and will be reported in due course.

## 3. Experimental Section

### 3.1. Chemistry

Melting points were obtained on a Büchi Melting Point B-540 apparatus (Büchi Labortechnik, Flawil, Switzerland) and were uncorrected. Mass spectra (MS) were taken in electrospray ionization (ESI) mode on an Agilent 1100 LC-MS (Agilent, Palo Alto, CA, USA). Proton (1H) nuclear magnetic resonance spectroscopy were performed using a Bruker ARX-300, 300 or 400 MHz spectrometers (Bruker Bioscience, Billerica, MA, USA) with tetramethylsilane (TMS) as an internal standard. Thin-layer chromatography (TLC) analysis was carried out on silica gel plates GF254 (Qingdao Haiyang Chemical, Qingdao, China). Unless otherwise noted, all common reagents and solvents were used as obtained from commercial suppliers without further purification. 

### 3.2. General Procedure for Preparation of 2-(4-Aminophenyl)-4-aminoquinazolines (***4a**–**b***)

Dioxane (100 mL) was added to a solution of sodium carbonate (3.2 g, 0.03 mol) in water (25 mL). Under argon, 2-chloro-4-aminoquinazoline **3** (0.01 mol), 4-aminophenylboronic acid pinacol ester (2.4 g, 0.011 mol) and Pd(PPh_3_)_2_Cl_2_ (0.7 g, 1 mmol) were added to the mixture successively. After refluxing for 6 h, water (50 mL) was added to the reaction mixture. The mixture was extracted by dichloromethane (50 mL × 3). The organic phase was washed by brine (50 mL × 1), dried, and evaporated to yield **4a**–**b**.

N^1^-(2-(4-aminophenyl)quinazolin-4-yl)-N^2^,N^2^-dimethylethane-1,2-diamine (**4a**). Yield: 73.5%; MS (ESI) *m*/*z*: 308.2 [M + H^+^]. 2-(4-Aminophenyl)-*N*-(3-morpholinopropyl)quinazolin-4-amine (**4b**). Yield: 78.1%; MS (ESI) *m*/*z*: 364.5 [M + H^+^].

### 3.3. General Procedure for Preparation of Componds ***5a**–**i***

A mixture of intermediate **4** (1 mmmol) and corresponding aromatic isocyanate **6** or isothiocyanate **7** (1.1 mmol) in dry THF (15 mL) was stirred at 30 °C for 6 h and monitored by TLC. The precipitate was collected by filtration, washed with ether, and purified by silica gel chromatography (MeOH:CH_2_Cl_2_ = 20:1) to afford target compounds **5a**–**i**.

*1-(2-Chloro-6-methylphenyl)-3-(4-(4-((2-(dimethylamino)ethyl)amino)quinazolin-2-yl)phenyl)urea* (**5a**). Yield: 62%; m.p.: 144.5–147.0 °C; MS (ESI) *m*/*z*: 474.9 [M + H^+^]; ^1^H-NMR (400 MHz, DMSO-*d*_6_) δ (ppm): 9.08 (s, 1H), 8.2.4 (d, *J* = 8.8 Hz, 2H ), 8.12 (t, *J* = 5.2, 4.8 Hz, H), 8.04 (d, *J* = 8.0 Hz, 1H), 7.95 (s, 1H), 7.56 (m, 2H), 7.45 (d, *J* = 8.4 Hz, 2H), 7.29 (m, 1H), 7.23 (d, *J* = 8.0 Hz, 1H), 7.11 (d, *J* = 7.6 Hz, 1H), 7.05 (m, 1H), 3.17 (q, 2H), 2.47 (t, *J* = 8.0 Hz, 2H), 2.38 (s, 3H), 2.21 (s, 6H). Anal. Calcd for C_26_H_27_ClN_6_O (%): C, 65.74; H, 5.73; N, 17.69; Found (%): C, 65.71; H, 5.76; N, 17.68.

*1-(4-(4-((2-(Dimethylamino)ethyl)amino)quinazolin-2-yl)phenyl)-3-(3,4-dimethylphenyl)urea* (**5b**). Yield: 68%; m.p.: 132.0–134.0 °C; MS (ESI) *m*/*z*: 455.2 [M + H^+^]; ^1^H-NMR (400 MHz, DMSO-*d*_6_) δ (ppm): 10.04 (s, 1H), 8.39 (t, *J* = 7.6 Hz, 1H), 8.24 (d, *J* = 7.2 Hz, 3H), 8.10 (d, *J* = 8.4 Hz, 1H), 7.55 (m, 3H), 7.46 (d, *J* = 8.4 Hz, 2H), 7.28 (t, *J* = 7.2 Hz, 1H), 6.89 (d, *J* = 7.2 Hz, 1H), 6.60 (d, *J* = 7.2 Hz, 1H), 3.17 (q, 2H), 2.47 (t, *J* = 8.0 Hz, 2H), 2.23 (s, 3H), 2.22 (s, 3H), 2.19 (s, 6H). Anal. Calcd for C_27_H_30_N_6_O (%): C, 71.34; H, 6.65; N, 18.49; Found (%): C, 71.36; H, 6.62; N, 18.47.

*1-(2-Chloro-6-methylphenyl)-3-(4-(4-((3-morpholinopropyl)amino)quinazolin-2-yl)phenyl)urea* (**5c**). Yield: 70%; m.p.: 152.5–154.5 °C; MS (ESI) *m*/*z*: 531.1 [M + H^+^]; ^1^H-NMR (400 MHz, DMSO-*d*_6_) δ (ppm): 9.20 (s, 1H), 8.38 (d, *J* = 8.4 Hz, 2H ), 8.28 (t, *J* = 4.8, 5.2 Hz, H), 8.18 (d, *J* = 8.4 Hz, 1H), 8.07 (s, 1H), 7.71 (m, 2H), 7.56 (d, *J* = 8.4 Hz, 2H), 7.42 (m, 1H), 7.35 (d, *J* = 7.6 Hz, 1H), 7.24 (d, *J* = 7.6 Hz, 1H), 7.19 (m, 1H), 3.70 (m, 2H), 3.57 (t, *J* = 8.4 Hz, 4H), 2.40 (m, 6H), 2.27 (s, 3H), 1.87(m, 2H). Anal. Calcd for C_29_H_31_ClN_6_O_2_ (%): C, 65.59; H, 5.88; N, 15.83; Found (%): C, 65.61; H, 5.85; N, 15.88.

*1-(3,4-Dimethylphenyl)-3-(4-(4-((3-morpholinopropyl)amino)quinazolin-2-yl)phenyl)urea* (**5d**). Yield: 56%; m.p.: 140.7–143.0 °C; MS (ESI) *m*/*z*: 510.9 [M + H^+^]; ^1^H-NMR (400 MHz, DMSO-*d*_6_) δ (ppm): 10.18 (s, 1H), 8.54 (t, *J* = 7.2 Hz, 1H), 8.37 (d, *J* = 7.2 Hz, 3H), 8.23 (d, *J* = 8.0 Hz, 1H), 7.69 (m, 3H), 7.59 (d, *J* = 8.0 Hz, 2H), 7.41 (t, *J* = 7.2 Hz, 1H), 7.01 (d, *J* = 7.2 Hz, 1H), 6.73 (d, *J* = 7.2 Hz, 1H), 3.70 (m, 2H), 3.57 (t, *J* = 7.2 Hz, 4H), 2.42 (t, *J* = 7.2 Hz, 2H), 2.37 (t, *J* = 7.2 Hz, 4H), 2.24 (s, 3H), 2.23 (s, 3H), 1.86 (m, 2H). Anal. Calcd for C_30_H_34_N_6_O_2_ (%): C, 70.56; H, 6.71; N, 16.46; Found (%): C, 70.55; H, 6.76; N, 16.42.

*1-(4-Fluorophenyl)-3-(4-(4-((3-morpholinopropyl)amino)quinazolin-2-yl)phenyl)urea* (**5e**). Yield: 52%; m.p.: 137.0–139.5 °C; MS (ESI) *m*/*z*: 501.2 [M + H^+^]; ^1^H-NMR (400 MHz, DMSO-*d*_6_) δ (ppm): 9.91 (s, 2H), 8.51 (t, *J* = 5.2 Hz, 1H), 8.39 (d, *J* = 8.8 Hz, 2H), 8.30 (d, *J* = 8.4 Hz, 1H), 7.72 (m, 4H), 7.60 (d, *J* = 8.8 Hz, 2H), 7.43 (m, 1H), 7.36 (d, *J* = 8.4 Hz, 2H), 4.15 (t, *J* = 6.8 Hz, 2H ), 3.72 (t, *J* = 6.4 Hz, 4H), 2.43 (t, *J* = 6.8 Hz, 2H), 2.38 (t, *J* = 6.4 Hz, 4H), 1.86 (m, 2H). Anal. Calcd for C_28_H_29_FN_6_O_2_ (%): C, 67.18; H, 5.84; N, 16.79; Found (%): C, 67.15; H, 5.88; N, 16.75.

*1-(4-Chlorophenyl)-3-(4-(4-((2-(dimethylamino)ethyl)amino)quinazolin-2-yl)phenyl)thiourea* (**5f**). Yield: 65%; m.p.: 131.5–134.0 °C; MS (ESI) *m*/*z*: 477.1 [M + H^+^]; ^1^H-NMR (400 MHz, DMSO-*d*_6_) δ (ppm): 10.75 (s, 2H), 8.31 (d, *J* = 8.4 Hz, 3H), 8.07 (d, *J* = 8.1 Hz, 1H), 7.68–7.59 (m, 4H), 7.45 (d, *J* = 8.4 Hz, 2H), 7.37–7.26 (m, 3H), 3.86–3.77 (q, 2H), 2.73 (t, *J* = 6.4 Hz, 2H), 2.33 (s, 6H). Anal. Calcd for C_25_H_25_ClN_6_S (%): C, 62.95; H, 5.28; N, 17.62; Found (%): C, 62.94; H, 5.27; N, 17.64.

*1-(4-(4-((2-(Dimethylamino)ethyl)amino)quinazolin-2-yl)phenyl)-3-(4-(trifluoromethoxy)phenyl)thiourea* (**5g**). Yield: 60%; m.p.: 150.8–153.0 °C; MS (ESI) *m*/*z*: 527.2 [M + H^+^]; ^1^H-NMR (400 MHz, DMSO-*d*_6_) δ (ppm): 10.12 (s, 1H), 10.03 (s, 1H), 8.25 (d, *J* = 8.4 Hz, 2H), 8.18 (t, *J* = 6.8 Hz, 1H), 8.05 (d, *J* = 8.0 Hz, 1H), 7.58 (m, 3H), 7.47 (d, *J* = 8.8 Hz, 2H), 7.31 (m, 3H), 6.96 (d, *J* = 8.4 Hz, 1H), 3.38 (q, 2H), 2.56 (t, *J* = 7.2 Hz, 2H), 2.23 (s, 6H). Anal. Calcd for C_26_H_25_F_3_N_6_OS (%): C, 59.30; H, 4.79; N, 15.96; Found (%): C, 59.32; H, 4.76; N, 15.94.

*1-(4-Chlorophenyl)-3-(4-(4-((3-morpholinopropyl)amino)quinazolin-2-yl)phenyl)thiourea* (**5h**). Yield: 63%; m.p.: 145.5–148.0 °C; MS (ESI) *m*/*z*: 533.8 [M + H^+^]; ^1^H-NMR (400 MHz, DMSO-*d*_6_) δ (ppm): 10.90 (s, 2H), 8.42 (d, *J* = 8.8 Hz, 3H), 8.24 (d, *J* = 8.4 Hz, 1H), 7.73 (m, 4H), 7.65 (d, *J* = 8.4 Hz, 2H), 7.44 (m, 1H), 7.38 (d, *J* = 8.8 Hz, 2H), 3.71 (m, 2H), 3.58 (t, *J* = 8.4 Hz, 4H), 2.43 (t, *J* = 7.2 Hz, 2H), 2.38 (t, *J* = 8.4 Hz, 4H), 1.88 (m, 2H). Anal. Calcd for C_28_H_29_ClN_6_OS (%): C, 63.09; H, 5.48; N, 15.76; Found (%): C, 63.05; H, 5.47; N, 15.79.

*1-(4-(4-((3-Morpholinopropyl)amino)quinazolin-2-yl)phenyl)-3-(4-(trifluoromethoxy)phenyl)thiourea* (**5i**). Yield: 59%; m.p.: 164.5–166.5 °C; MS (ESI) *m*/*z*: 583.9 [M + H^+^]; ^1^H-NMR (400 MHz, DMSO-*d*_6_) δ (ppm): 10.25 (s, 1H), 10.16 (s, 1H), 8.45 (d, *J* = 8.8 Hz, 2H), 8.35 (t, *J* = 7.2 Hz, 1H), 8.22 (d, *J* = 8.4 Hz, 1H), 7.74 (m, 3H), 7.64 (d, *J* = 8.8 Hz, 2H), 7.48 (m, 3H), 7.11 (d, *J* = 8.0 Hz, 1H), 3.73 (m, 2H), 3.59 (t, *J* = 4.4 Hz, 4H), 2.44 (t, *J* = 7.2 Hz, 2H), 2.39 (t, *J* = 4.4 Hz, 4H), 1.89 (m, 2H). Anal. Calcd for C_29_H_29_F_3_N_6_O_2_S (%): C, 59.78; H, 5.02; N, 14.42; Found (%): C, 59.75; H, 5.01; N, 14.47.

### 3.4. General Procedure for Preparation of Aromatic Isocyanates (***6a**–**n***)

Appropriate aromatic amine (0.02 mol) was added slowly to a stirred solution of BTC (3.0 g, 0.01 mol) in 1,4-dioxane (30 mL) at room temperature. After refluxing for 8-12 h, excess 1,4-dioxane was evaporated. The residue was distilled under reduced pressure to afford compounds **6a**–**n**.

*1-Chloro-4-isocyanatobenzene* (**6a**). Purity: 78.2%; b.p.: 110–114 °C (30–40 mmHg).

*4-Isocyanato-1,2-dimethylbenzene* (**6b**). Purity: 80.5%; b.p.: 59–65 °C (30–40 mmHg).

*1-Chloro-2-isocyanato-3-methylbenzene* (**6c**). Purity: 80.8%; b.p.: 116–118 °C (30–40 mmHg).

*1-Fluoro-4-isocyanatobenzene* (**6d**). Purity: 84.2%; b.p.: 94–95 °C (30–40 mmHg).

*1-Fluoro-3-isocyanatobenzene* (**6e**). Purity: 83.6%; b.p.: 85–91 °C (30–40 mmHg).

*1-Fluoro-2-isocyanatobenzene* (**6f**). Purity: 84.3%; b.p.: 76–80 °C (30–40 mmHg).

*1-Chloro-3-isocyanatobenzene* (**6g**). Purity: 80.1%; b.p.: 125–128 °C (30–40 mmHg).

*2-Chloro-1-fluoro-4-isocyanatobenzene* (**6h**). Purity: 85.2%; b.p.: 93–98 °C (30–40 mmHg).

*1,3-Difluoro-2-isocyanatobenzene* (**6i**). Purity: 80.9%; b.p.: 67–71 °C (30–40 mmHg).

*2-Isocyanato-1,3-dimethylbenzene* (**6j**). Purity: 76.9%; b.p.: 67–71 °C (30–40 mmHg).

*1-Isocyanato-2,4-dimethylbenzene* (**6k**). Purity: 77.5%; b.p.: 71–77 °C (30–40 mmHg).

*1-Isocyanato-3-(trifluoromethyl)benzene* (**6l**). Purity: 83.2%; b.p.: 105–111 °C (30–40 mmHg).

*1-Isocyanato-4-(trifluoromethoxy)benzene* (**6m**). Purity: 81.0%; b.p.: 113–116 °C (30–40 mmHg).

*Isocyanatobenzene* (**6n**). Purity: 80.2%; b.p.: 45–50 °C (30–40 mmHg).

### 3.5. General Procedure for Preparation of Aromatic Isothiocyanates (***7a**,**b***)

Appropriate aromatic amine (0.02 mol) and DABCO (6.7 g, 0.06 mol) were dissolved in toluene (25 mL) at room temperature. Under stirring, CS_2_ (4.6 g, 0.06 mol) was added dropwise to the solution within 20 min. After stirring at room temperature for 12 h, the precipitate was collected by filtration and washed with toluene. The residue was dried and suspended in CHCl_3_ (25 mL), and BTC (19.6 g, 0.066 mol) in CHCl_3_ (25 mL) was added dropwise to the mixture slowly in an ice bath. Then the mixture was stirred for 1 h at room temperature and refluxed for 1 h. The mixture was filtered and evaporated to give compounds **7a**,**b**.

*1-Chloro-4-isothiocyanatobenzene* (**7a**). Purity: 77.5%. 

*1-Isothiocyanato-4-(trifluoromethoxy)benzene* (**7b**). Purity: 79.8%.

### 3.6. Ethyl 4-aminobenzimidate Dihydrochloride (***8***)

Dry hydrogen chloride was bubbled into the mixture of 4-aminobenzonitrile (15 g, 0.127 mol), 1,4-dioxane (100 mL) and anhydrous ethanol (100 mL) for 6 h in an ice bath. Then the mixture was sealed and stirred at room temperature for 48 h. After completion, the solution was concentrated under reduced pressure and the semisolid residue was diluted with anhydrous ether (200 mL). The suspension was filtered, washed with anhydrous ether and dried to give the title compound **8** as a white solid (29.3 g, 97.7%). MS (ESI) *m*/*z*: 164.9 [M + H^+^].

### 3.7. 4-Aminobenzimidamide Hydrochloride (***9***)

Dry ammonia was bubbled into the mixture of compound **8** (29.3 g, 0.123 mol) and anhydrous ethanol (200 mL) for 6 h in an ice bath. Then the mixture was sealed and stirred at room temperature for 24 h. Then the solution was concentrated under a reduced pressure and the semisolid residue was diluted with anhydrous ether (200 mL). The suspension was filtered, washed with anhydrous ether, and dried to give the title compound **9** as a white solid (20.2 g, 95.3%). MS (ESI) *m*/*z*: 135.9 [M + H^+^].

### 3.8. 3-(Dimethylamino)-2-(pyridin-2-yl)acrylonitrile (***10***)

A mixture of 2-(pyridin-2-yl)acetonitrile (15.0 g, 0.127 mol), DMF-DMA (30 mL, 0.229 mol) and methanol (36 mL) was stirred at 30 °C for 24 h. The solvent was evaporated, moderate methanol and ethyl acetate was added in. The solution was concentrated under a reduced pressure to remove excess DMF-DMA completely. The residue was poured into petroleum ether (100 mL) and stirred for 6 h. The suspension was separated by filtration and washed with ether to give compound **10** as a dark red solid (18.3 g, 83.2%). MS (ESI) *m*/*z*: 174.0 [M + H^+^].

### 3.9. 2-(4-Aminophenyl)-5-(pyridin-2-yl)pyrimidin-4-amine (***11***)

A mixture of compound **9** (20.2 g, 0.118 mol ), compound **10** (17.0 g, 0.098 mol), sodium carbonate (12.5 g, 0.0118 mol), methnol (200 mL) and water (60 mL) was heated to 70 °C for 24 h. The solution was concentrated and added to water (200 mL). The precipitate was collected by filtration, washed with ether, and recrystallized with ethnol to afford compound 11 as a pale white solid (13.9 g, 53.8%). MS (ESI) *m*/*z*: 264.1 [M + H^+^].

### 3.10. General Procedure for Preparation of Compounds ***12a**–**l*** and ***13a**–**l***

A mixture of intermediate **11** (1 mmmol) and corresponding aromatic isocyanate **6** (1.1 mmol) in dry THF (15 mL) was stirred at 30 °C for 6 h and monitored by TLC. The precipitate was collected by filtration, washed with ether, and dried to afford compounds **12a**–**l** without further purification. At room temperature, 20%–30% hydrochloride ethanol solution (1 mL) was added dropwise to a stirred solution of compound **12** dissolved in CHCl_3_ (10 mL). The suspension was stirred for 1 h, filtered and dried to give the corresponding compounds **13a**–**l**.

*1-(4-(4-Amino-5-(pyridin-2-yl)pyrimidin-2-yl)phenyl)-3-phenylurea dihydrochloride* (**13a**). Yield: 79%; m.p.: 303.0–306.0 °C; MS (ESI) *m*/*z*: 383.1 [M + H^+^]; ^1^H-NMR (300 MHz, DMSO-*d*_6_) δ (ppm): 9.41 (s, 1H), 9.11 (s, 1H), 8.93 (s, 2H), 8.77 (s, 1H), 8.68 (d, *J* = 4.4 Hz, 1H), 8.32 (d, *J* = 8.4 Hz, 2H), 8.10 (d, *J* = 8.0 Hz, 1H), 7.94 (t, *J* = 7.9 Hz, 1H), 7.58 (d, *J* = 8.5 Hz, 2H), 7.48 (d, *J* = 8.4 Hz), 7.38–7.32 (m, 1H), 7.30 (t, *J* = 7.7 Hz, 2H), 7.00 (t, *J* = 6.9 Hz). Anal. Calcd for C_22_H_20_C_l2_N_6_O (%): C, 58.03; H, 4.43; N, 18.46; Found (%): C, 58.07; H, 4.46; N, 18.42.

*1-(4-(4-Amino-5-(pyridin-2-yl)pyrimidin-2-yl)phenyl)-3-(2,4-dimethylphenyl)urea dihydrochloride* (**13b**). Yield: 65%; m.p.: 286.0–288.5 °C; MS (ESI) *m*/*z*: 411.3 [M + H^+^]; ^1^H-NMR (300 MHz, DMSO-*d*_6_) δ (ppm): 9.77 (s, 1H), 8.89 (s, 1H), 8.74 (d, *J* = 4.6 Hz, 1H), 8.25(d, *J* = 8.6 Hz, 2H), 8.22 (d, *J* = 7.8 Hz, 1H), 8.05 (t, *J* = 7.9 Hz, 1H), 7.72(d, *J* = 8.6 Hz, 2H), 7.66 (d, *J* = 8.1 Hz, 1H), 7.52 (t, *J* = 4.8 Hz, 1H), 7.02 (s, 1H), 6.98 (d, *J* = 8.2 Hz, 2H), 2.24 (s, 6H). Anal. Calcd for C_24_H_24_C_l2_N_6_O (%): C, 59.63; H, 5.00; N, 17.39; Found (%): C, 59.60; H, 5.06; N, 17.34.

*1-(4-(4-Amino-5-(pyridin-2-yl)pyrimidin-2-yl)phenyl)-3-(6-chloro-2-methylphenyl)urea dihydrochloride* (**13c**). Yield: 72%; m.p.: 252.0–254.0 °C; MS (ESI) *m*/*z*: 431.0 [M + H^+^]; ^1^H-NMR (300 MHz, DMSO-*d*_6_) δ (ppm): 9.78 (s, 1H), 8.85 (s, 1H), 8.74 (d, *J* = 4.2 Hz, 1H), 8.56 (s, 1H), 8.26 (d, *J* = 8.7 Hz, 2H), 8.22 (d, *J* = 8.1 Hz, 1H), 8.04 (t, *J* = 6.7 Hz, 1H), 7.72 (d, *J* = 8.9 Hz, 2H), 7.54–7.50 (m, 1H), 7.36 (d, *J* = 5.4 Hz, 1H), 7.27–7.18 (m, 2H), 2.28 (s, 3H). Anal. Calcd for C_23_H_21_Cl_3_N_6_O (%): C, 54.83; H, 4.20; N, 16.68; Found (%): C, 54.84; H, 4.23; N, 16.65.

*1-(4-(4-Amino-5-(pyridin-2-yl)pyrimidin-2-yl)phenyl)-3-(2,6-dimethylphenyl)urea dihydrochloride* (**13d**). Yield: 76%; m.p.: 271.0–274.0 °C; MS (ESI) *m*/*z*: 411.2 [M + H^+^]; ^1^H-NMR (300 MHz, DMSO-*d*_6_) δ (ppm): 9.83 (s, 1H), 8.85 (s, 1H), 8.74(d, *J* = 4.2 Hz, 1H), 8.34 (s, 1H), 8.23 (d, *J* = 9.3 Hz, 2H), 8.21 (d, *J* = 8.1 Hz, 1H), 8.05(t, *J* = 7.8 Hz, 1H), 7.72 (d, *J* = 9.0 Hz, 2H), 7.54–7.50 (m, 1H), 7.08 (s, 3H), 2.23 (s, 6H). Anal. Calcd for C_24_H_24_C_l2_N_6_O (%): C, 59.63; H, 5.00; N, 17.39; Found (%): C, 59.67; H, 5.02; N, 17.36.

*1-(4-(4-Amino-5-(pyridin-2-yl)pyrimidin-2-yl)phenyl)-3-(2,6-difluorophenyl)urea dihydrochloride* (**13e**). Yield: 68%; m.p.: 279.5–281.0 °C; MS (ESI) *m*/*z*: 419.1 [M + H^+^]; ^1^H-NMR (300 MHz, DMSO-*d*_6_) δ (ppm): 9.78 (s, 1H), 8.88 (s, 1H), 8.73 (d, *J* = 4.7 Hz, 1H), 8.53 (s, 1H), 8.40 (s, 2H), 8.24 (d, *J* = 8.8 Hz, 2H), 8.20 (d, *J* = 9.3 Hz, 1H), 8.04 (t, *J* = 7.8 Hz, 1H), 7.72 (d, *J* = 8.6 Hz, 2H), 7.54–7.49 (m, 1H), 7.32–7.25 (m, 3H). Anal. Calcd for C_22_H_18_C_l2_F_2_N_6_O (%): C, 53.78; H, 3.69; N, 17.10; Found (%): C, 53.75; H, 3.72; N, 17.08.

*1-(4-(4-Amino-5-(pyridin-2-yl)pyrimidin-2-yl)phenyl)-3-(3-chloro-4-fluorophenyl)urea dihydrochloride* (**13f**). Yield: 80%; m.p.: 289.5–291.5 °C; MS (ESI) *m*/*z*: 435.0 [M + H^+^]; ^1^H-NMR (300 MHz, DMSO-*d*_6_) δ (ppm): 9.69 (s, 1H), 9.55 (s, 1H), 8.88 (s, 1H), 8.74 (d, *J* = 4.4 Hz, 1H), 8.25 (d, *J* = 8.8 Hz, 2H), 8.21 (d, *J* = 8.2 Hz, 1H), 8.05 (t, *J* = 7.8 Hz, 1H), 7.85–7.82 (m, 1H), 7.72 (d, *J* = 8.8 Hz, 2H), 7.54–7.49 (m, 1H), 7.40–7.34 (m, 2H). Anal. Calcd for C_22_H_18_C_l3_FN_6_O (%): C, 52.04; H, 3.57; N, 16.55; Found (%): C, 52.05; H, 3.52; N, 16.56.

*1-(4-(4-Amino-5-(pyridin-2-yl)pyrimidin-2-yl)phenyl)-3-(3-chlorophenyl)urea dihydrochloride* (**13g**). Yield: 71%; m.p.: 270.0–272.5 °C; MS (ESI) *m*/*z*: 417.0 [M + H^+^]; ^1^H-NMR (300 MHz, DMSO-*d*_6_) δ (ppm): 9.89 (s, 1H), 9.71 (s, 1H), 8.86 (s, 1H), 8.74 (d, *J* = 4.5 Hz, 1H), 8.25 (d, *J* = 8.7 Hz, 2H), 8.21 (d, *J* = 8.2 Hz, 1H), 8.05 (t, *J* = 7.9 Hz, 1H), 7.73 (d, *J* = 8.8 Hz, 2H), 7.72 (s, 1H), 7.55–7.51 (m, 1H), 7.33–7.30 (m, 2H), 7.04 (d, *J* = 5.8 Hz, 1H). Anal. Calcd for C_22_H_19_C_l3_N_6_O (%): C, 53.95; H, 3.91; N, 17.16; Found (%): C, 53.94; H, 3.92; N, 17.14.

*1-(4-(4-Amino-5-(pyridin-2-yl)pyrimidin-2-yl)phenyl)-3-(4-(trifluoromethyl)phenyl)urea dihydrochloride* (**13h**). Yield: 65%; m.p.: 267.0–269.5 °C; MS (ESI) *m*/*z*: 451.1 [M + H^+^]; ^1^H-NMR (300 MHz, DMSO-*d*_6_) δ (ppm): 9.78 (s, 1H), 9.74 (s, 1H), 8.89 (s, 1H), 8.74 (d, *J* = 4.5 Hz, 1H), 8.26 (d, *J* = 8.8 Hz, 2H), 8.21 (m, 2H), 8.05 (s, 2H), 7.74 (d, *J* = 8.8 Hz, 2H), 7.61–7.50 (m, 4H), 7.35 (d, *J* = 7.6 Hz, 1H). Anal. Calcd for C_23_H_19_C_l2_F_3_N_6_O (%): C, 52.79; H, 3.66; N, 16.06; Found (%): C, 52.75; H, 3.69; N, 16.05.

*1-(4-(4-Amino-5-(pyridin-2-yl)pyrimidin-2-yl)phenyl)-3-(4-(trifluoromethoxy)phenyl)urea dihydrochloride* (**13i**). Yield: 78%; m.p.: 308.5–310.5 °C; MS (ESI) *m*/*z*: 467.1 [M + H^+^]; ^1^H-NMR (300 MHz, DMSO-*d*_6_) δ (ppm): 9.50 (s, 1H), 9.35 (s, 1H), 8.89 (s, 1H), 8.74 (d, *J* = 4.5 Hz, 1H), 8.24 (d, *J* = 8.7 Hz, 2H), 8.19 (d, *J* = 8.4 Hz, 1H), 8.04 (t, *J* = 6.7 Hz, 1H), 7.71 (d, *J* = 8.7 Hz, 2H), 7.59 (d, 2H), 7.60–7.51 (m, 1H), 7.32 (d, *J* = 8.1 Hz, 2H). Anal. Calcd for C_23_H_19_C_l2_F_3_N_6_O_2_ (%): C, 51.22; H, 3.55; N, 15.58; Found (%): C, 51.24; H, 3.59; N, 15.54.

*1-(4-(4-Amino-5-(pyridin-2-yl)pyrimidin-2-yl)phenyl)-3-(4-fluorophenyl)urea dihydrochloride* (**13j**). Yield: 75%; m.p.: 264.0–267.0 °C; MS (ESI) *m*/*z*: 401.0 [M + H^+^]; ^1^H-NMR (300 MHz, DMSO-*d*_6_) δ (ppm): 9.80 (s, 1H), 9.47 (s, 1H), 8.87 (s, 1H), 8.75 (d, *J* = 4.6 Hz, 1H), 8.26 (d, *J* = 8.7 Hz, 2H), 8.22 (d, *J* = 7.8 Hz, 1H), 8.07 (t, *J* = 7.9 Hz, 1H), 7.73 (d, *J* = 8.8 Hz, 2H), 7.56–7.52 (m, 2H), 7.49 (t, *J* = 4.8 Hz, 1H), 7.15 (t, *J* = 8.8 Hz, 2H). Anal. Calcd for C_22_H_19_C_l2_FN_6_O (%): C, 55.82; H, 4.05; N, 17.76; Found (%): C, 55.84; H, 4.01; N, 17.79.

*1-(4-(4-Amino-5-(pyridin-2-yl)pyrimidin-2-yl)phenyl)-3-(3-fluorophenyl)urea dihydrochloride* (**13k**). Yield: 67%; m.p.: 228.5–231.0 °C; MS (ESI) *m*/*z*: 401.1 [M + H^+^]; ^1^H-NMR (300 MHz, DMSO-*d*_6_) δ (ppm): 9.89 (s, 1H), 9.73 (s, 1H), 8.86 (s, 1H), 8.74 (d, *J* = 4.5 Hz), 8.26 (d, *J* = 8.9 Hz, 2H), 8.22 (d, *J* = 8.4 Hz, 1H), 8.05 (t, *J* = 7.8 Hz, 1H), 7.72 (d, *J* = 8.9 Hz, 2H), 7.55 (s, 1H), 7.53–7.50 (m, 1H), 7.37–7.29 (m, 1H), 7.14 (d, *J* = 8.1 Hz, 1H), 6.82 (t, *J* = 8.4 Hz, 1H). Anal. Calcd for C_22_H_19_C_l2_FN_6_O (%): C, 55.82; H, 4.05; N, 17.76; Found (%): C, 55.81; H, 4.06; N, 17.72.

*1-(4-(4-Amino-5-(pyridin-2-yl)pyrimidin-2-yl)phenyl)-3-(2-fluorophenyl)urea dihydrochloride* (**13l**). Yield: 73%; m.p.: 258.5–261.5 °C; MS (ESI) *m*/*z*: 401.0 [M + H^+^]; ^1^H-NMR (300 MHz, DMSO-*d*_6_) δ (ppm): 10.01 (s, 1H), 8.94 (s, 1H), 8.87 (s, 1H), 8.74 (d, *J* = 4.5 Hz, 1H), 8.27 (d, *J* = 9.0 Hz, 2H), 8.22 (d, *J* = 8.1 Hz, 1H), 8.16–8.03 (m, 1H), 7.74 (d, *J* = 8.7 Hz, 2H), 7.55–7.50 (m, 1H), 7.29–7.03 (m, 3H). Anal. Calcd for C_22_H_19_C_l2_FN_6_O (%): C, 55.82; H, 4.05; N, 17.76; Found (%): C, 55.86; H, 4.03; N, 17.75.

### 3.11. Evaluation of the Biological Activity

The antitumor activity of compounds **5a**–**i** and **13a**–**l** was evaluated with HT-29, H-460, A549, and MDA-MB-231 by the MTT method in vitro, with sorafenib as positive control. The cancer cells were cultured in minimum essential medium (MEM) supplemented with 10% fetal bovine serum (FBS).

Approximately 4 × 10^3^ cells, suspended in MEM medium, were plated onto each well of a 96-well plate and incubated in 5% CO_2_ at 37 °C for 24 h. The test compounds were added to the culture medium at the indicated final concentrations and the cell cultures were continued for 72 h. Fresh MTT was added to each well at a final concentration of 5 μg/mL and incubated with cells at 37 °C for 4 h. The formazan crystals were dissolved in 100 μL DMSO per each well, and the absorbency at 492 nm (for the absorbance of MTT formazan) and 630 nm (for the reference wavelength) was measured with the ELISA reader. All of the compounds were tested twice in each of the cell lines. The results expressed as IC_50_ (inhibitory concentration of 50%) were the averages of two determinations and were calculated by using the Bacus Laboratories Incorporated Slide Scanner (Bliss) software.

### 3.12. EGFR and VEGFR2/KDR Kinases Assay In Vitro

The target compound **5a** was tested for its activity against EGFR and VEGFR2/KDR kinases through the mobility shift assay. All kinase assays were performed in 96-well plates in a 50 μL reaction volume. The kinase buffer contains 50 mM HEPES, pH 7.5, 10 mM MgCl_2_, 0.0015% Brij-35 and 2 mM DTT. The stop buffer contains 100 mM HEPES, pH 7.5, 0.015% Brij-35, 0.2% Coating Reagent #3, and 50 mM ethylene diamine tetraacetic acid (EDTA). Compounds were diluted to 500 μM by 100% DMSO, then 10 μL of the compounds were transfered to a new 96-well plate as the intermediate plate, and 90 μL kinase buffer was added to each well. Five microliters of each well of the intermediate plate was transferred to a 384-well plate. The following amounts of enzyme and substrate were used per well: kinase base buffer, FAM-labeled peptide, ATP, and enzyme solution. Wells containing the substrate, enzyme, and DMSO without compound were used as the DMSO control. Wells containing just the substrate without enzyme were used as the low control. The compounds were incubated at room temperature for 10 min. Ten microliters of peptide solution was added to each well and incubated at 28 °C for a specified period of time and the reaction stopped by 25 μL of stop buffer. Finally, data was collected using the Caliper program, which converted conversion values to inhibition values.

Percent inhibition = (max − conversion)/(max − min) × 100,(1)

where “max” stands for DMSO control; “min” stands for low control.

## 4. Conclusions

Taken as a whole, two novel series of diaryl urea derivatives were synthesized and evaluated for their cytotoxicity against four human cancer cell lines (H-460, HT-29, A549, and MDA-MB-231). Our strategy that 4-aminoquinazolinyl moiety was incorporated into the diaryl urea scaffold conveyed cellular potency and resulted in analogues **5a**–**i** demonstrating excellent activity in the single-digit μM range, superior to sorafenib. The preliminary SARs showed that electrophilic groups on terminal phenyl ring and steric hindrance in ortho-position were beneficial to improved antitumor potency. The exploration of detailed SARs led to the identification of EGFR inhibitor **5a** as a valuable lead, which possessed prominent activity both in cellular (IC_50_ = 0.15, 0.089, 0.36, and 0.75 μM, respectively) and enzymatic assay (IC_50_ = 56 nM against EGFR). Further optimization and investigation based on these structures are in progress. 

## Figures and Tables

**Table 1:** Structures and cytotoxicity of compounds **5a**–**i** against HT-29, H-460, A-549, and MDA-MB-231 cells in vitro.

Compd.	X	n	R^2^	R^1^	IC_50_ (μM) ^a^			
					HT-29	H-460	A-549	MDA-MB-231
**5a**	O	2	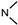	2-Cl, 6-CH_3_	0.15 ± 0.14	0.089 ± 0.11	0.36 ± 0.27	0.75 ± 0.65
**5b**	O	2	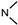	3,4-(CH_3_)_2_	1.09 ± 0.21	1.50 ± 0.13	2.33 ± 1.52	2.42 ± 0.28
**5c**	O	3	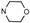	2-Cl, 6-CH_3_	0.36 ± 0.72	0.27 ± 0.12	0.82 ± 0.43	1.08 ± 0.15
**5d**	O	3	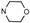	3,4-(CH_3_)_2_	1.72 ± 0.37	2.93 ± 0.62	5.46 ± 0.38	3.92 ± 1.24
**5e**	O	3	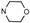	4-F	2.64 ± 0.53	1.19 ± 0.96	3.41 ± 1.27	2.72 ± 0.42
**5f**	S	2	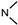	4-Cl	0.24 ± 0.17	0.77 ± 0.22	2.30 ± 0.18	2.59 ± 0.36
**5g**	S	2	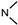	4-OCF_3_	1.05 ± 0.012	1.28 ± 0.68	2.81 ± 0.73	3.02 ± 0.51
**5h**	S	3	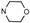	4-Cl	0.65 ± 0.33	1.02 ± 0.74	1.44 ± 0.39	3.13 ± 0.85
**5i**	S	3	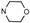	4-OCF_3_	2.41 ± 0.21	2.58 ± 0.74	4.86 ± 0.56	4.07 ± 1.02
**Sorafenib**					3.27 ± 0.32	2.15 ± 0.43	4.47± 0.28	3.81± 0.50

^a^ Results are expressed as means ± SD (standard deviation) of three independent experiments.

**Table 2:** Structures and cytotoxicity of compounds **13a**–**l** against HT-29, H-460, A-549, and MDA-MB-231 cells in vitro.

Compd.	R^1^	IC_50_ (μM) ^a^			
		HT-29	H-460	A549	MDA-MB-231
**13a**	H	30.52 ± 2.56	28.64 ± 1.08	44.83 ± 2.42	32.16 ± 1.59
**13b**	2,4-diCH_3_	45.08 ± 1.37	NA	NA	NA
**13c**	2-Cl, 6-CH_3_	12.95 ± 0.68	24.66 ± 0.80	30.71 ± 1.73	25.42 ± 1.14
**13d**	2,6-diCH_3_	18.37 ± 0.45	24.62 ± 2.75	15.94 ± 0.95	28.12 ± 2.44
**13e**	2,6-diF	35.10 ± 2.81	ND	NA	20.94 ± 1.63
**13f**	3-Cl, 4-F	6.33 ± 0.93	15.28 ± 1.19	10.49 ± 2.26	12.75 ± 2.97
**13g**	3-Cl	10.07 ± 0.32	5.86 ± 1.34	15.43 ± 1.02	21.95 ± 2.58
**13h**	3-CF_3_	ND	ND	ND	ND
**13i**	4-OCF_3_	26.58 ± 1.27	23.46 ± 2.29	16.54 ± 0.70	30.65 ± 3.06
**13j**	4-F	17.95 ± 0.94	32.28 ± 2.87	ND	NA
**13k**	3-F	30.50 ± 3.16	ND	NA	NA
**13l**	2-F	24.18 ± 1.35	32.29 ± 2.63	NA	35.56 ± 2.85
**Sorafenib**		3.27 ± 0.32	2.15 ± 0.43	4.47 ± 0.28	3.81 ± 0.50

^a^ Results are expressed as means ± SD (standard deviation) of three independent experiments. NA: compound showing IC_50_ value > 50 μM. ND: Not determined.

**Table 3:** EGFR and VEGFR2/KDR kinases inhibitory activity of compound **5a** in vitro.

Compd.	IC_50_ (nM)	
	VEGFR2/KDR	EGFR
**5a**	>3000	56
**Sorafenib**	93	-
